# Effects of normobaric hypoxic endurance training on fatigue in patients with multiple sclerosis: a randomized prospective pilot study

**DOI:** 10.1007/s00415-021-10596-5

**Published:** 2021-05-18

**Authors:** Tobias Zrzavy, Anna Pfitzner, Peter Flachenecker, Paulus Rommer, Uwe Klaus Zettl

**Affiliations:** 1 Department of Neurology, Medical University of Vienna, Waehringer Guertel 18-20, 1090 Vienna, Austria; 2 Department of Neurology, Rostock University Medical Center, Rostock, Germany; 3 Department of Nephrology and Endocrinology/Diabetology, Klinikum Ernst von Bergmann, Potsdam, Germany; 4 Neurological Rehabilitation Center Quellenhof, Bad Wildbad, Germany

**Keywords:** Multiple sclerosis, Fatigue, Endurance training, Hypoxic, Rehabilitation

## Abstract

**Background:**

Fatigue is one of the most frequent symptoms in patients with multiple sclerosis (MS), causing a major impact on quality-of-life. Non-pharmacological intervention strategies involve physical activity, which has been shown to reduce fatigue. Training under normobaric hypoxic conditions is thought to improve the response to endurance training and may, therefore, have an additional benefit over normoxic training conditions in MS patients.

**Objective:**

To compare the effects of endurance training under hypoxic and normoxic conditions on fatigue, mobility and spasticity in patients with MS during inpatient rehabilitation.

**Methods:**

Thirty-nine patients with MS were assigned within a randomized prospective longitudinal pilot study to (1) a routine clinical rehabilitation program, (2) a routine clinical rehabilitation program + normoxic endurance training and (3) a routine clinical rehabilitation program + hypoxic endurance training for 14 days. Fatigue (WEIMuS and MFIS), spasticity (MSSS-88) and walking endurance (6MinWT) were assessed at days 0, 7 and 14.

**Results:**

Fatigue scores improved significantly in all groups, but these improvements were reached faster in the groups which additionally received endurance training (normoxic *p* = 0.004; hypoxic *p* = 0.002). Spasticity scores were significantly lower in endurance training groups at the end of the study compared to baseline (normoxic *p* = 0.048, hypoxic *p* = 0.012), while only the hypoxic group increased significantly in 6MinWT (*p* = 0.001).

**Conclusions:**

Our findings demonstrate that endurance training provides substantial benefit to neurological rehabilitation programs. Endurance training under hypoxic conditions could positively influence walking endurance within a 2-week training intervention and warrants further investigations.

## Introduction

Multiple sclerosis (MS) is an immune-mediated and neurodegenerative disease of the central nervous system (CNS) that is clinically heterogeneous and the most common cause of neurological disability in young adults [3, 25, 28]. One of the most frequent symptoms, namely fatigue, has a major impact on quality-of-life in affected individuals [19, 24, 26]. To date, the underlying pathophysiology of MS-related fatigue is far from understood [14]. Inflammation associated release of cytokines, disturbance of the hypothalamus-pituitary axis, as well as axonal damage to the CNS leading to compensatory increased activation of brain regions for handling cognitive and motoric tasks are hypothesized. So far, treatment options are limited and evidence for pharmacological therapy in improving MS-related fatigue is considered to be rather low [17, 27].

One of the most efficacious non-pharmacological intervention strategies is physical activity, which has been numerously shown to reduce fatigue in MS patients, and therefore, is recommended as important treatment option [8]. Although there is strong evidence for exercise as an overall beneficial rehabilitation strategy in MS, individuals with MS are less active than the non-diseased population [15].

Since the Olympic games in Mexico in 1968, the effects of training in altitudes got increasing interest in many studies. Nowadays, the performance-enhancing effect is undisputed and training under normobaric hypoxic conditions is implied to improve response to endurance training at a lower intensity compared to normoxic conditions, and therefore, might be a beneficial training option also for MS patients [7]. However, the effects of hypoxic training conditions on MS have only been investigated in one study [13]. This pilot study aimed to investigate the effects of endurance training under hypoxic conditions on fatigue, mobility, and spasticity in people with MS and whether this form of training might be suitable to be implemented in routine rehabilitation of MS patients.

## Patients and methods

The ethics committee of the State Chamber of Physicians of Baden-Württemberg approved the study (2007-123-F). Written consent was obtained from all patients prior to the study. The study has been registered at the German Clinical Trials Register (Study identifier Nr: DRKS00006307).

**Fig. 1:**
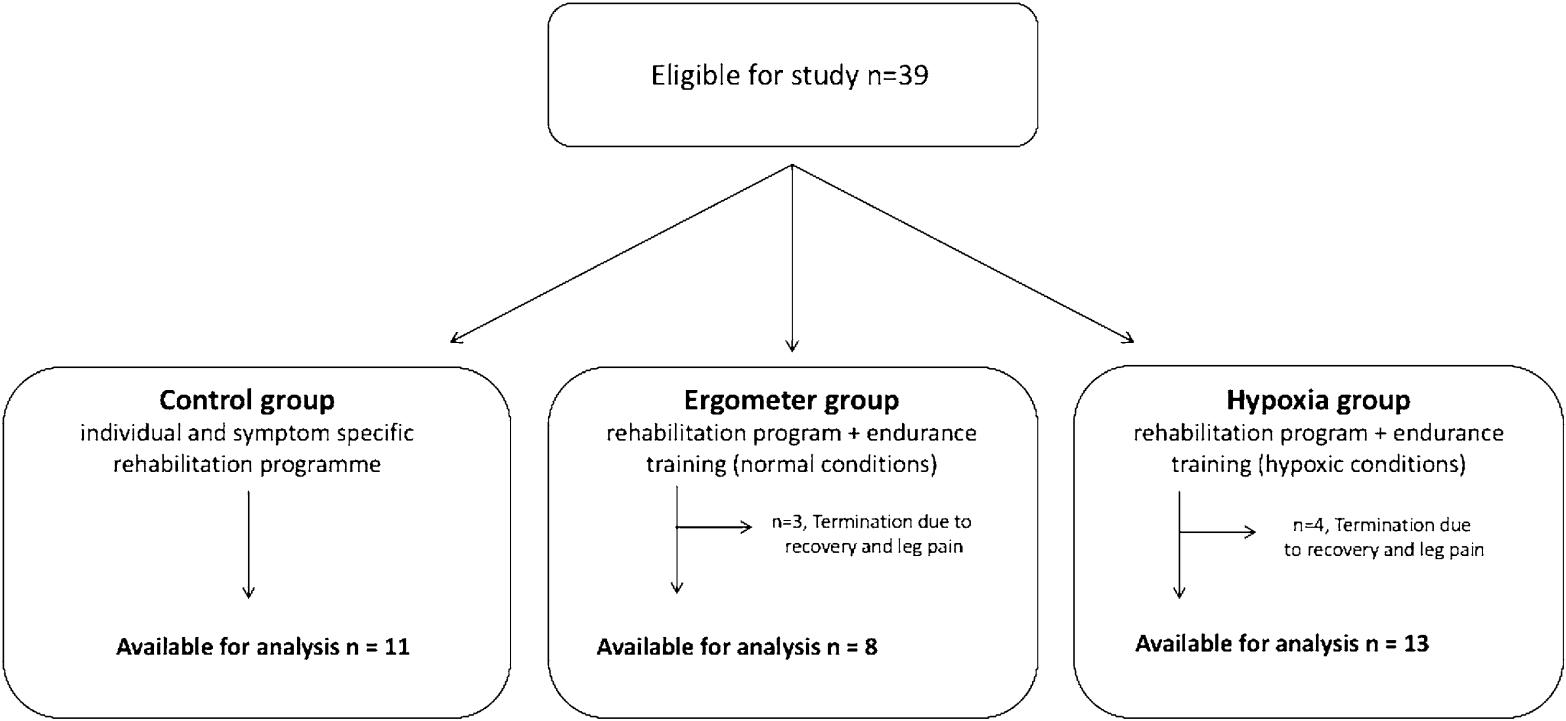
Inclusion flowchart

This monocentric randomized prospective longitudinal pilot study enrolled 39 patients with MS assigned to an inpatient rehabilitation center during the study period (14.07.2008–14.10.2008) (Fig. 1). Randomized allocation of patients to either of the groups was based on drawing pre-generated lots. Participants and investigators were blinded to the group allocation. Since the training supervisors had to determine the conditions for the decompression chamber, they were aware of group allocation.

The inclusion criteria were (1) clinically definite MS—relapsing or progressive—according to the McDonald criteria [20, 21], (2) EDSS ≤ 6.0, (3) age > 18 years and (4) self-reported fatigue.

Exclusion criteria were: (1) serious cardiovascular or pulmonary diseases based on the assessment of specialists in internal medicine, (2) relapse or treatment with glucocorticosteroids 30 days prior to screening, (3) severe spasticity or other impairment (mobility restriction or cognitive decline) that made it impossible to perform the tasks within the study, and (4) disease-modifying therapy change. After written informed consent, the patients were randomly assigned to one of three groups (Fig. 1): all patients received their individual, symptom-specific and goal-oriented rehabilitation program. Group I (Control) only received this program; group II (Ergometer group) received an additional endurance training (exercise bike, 12 times for 45 min over 12 days, separated by a day without training) under normal conditions, and group III (Hypoxia group) received this endurance training (exercise bike, 12 times for 45 min over 12 days, separated by a day without training) under hypoxic conditions (decompression chamber).

### Endurance training

The training consisted of a 45-min bicycle ergometer session (Ergo-Fit Cardioline 400 Med) with three 15-min training intervals separated by a 5-min break between each interval. Pulse and wattage were monitored and recorded for each 15-min block. The pedaling frequency was standardized to 60 rotations per min. Aerobe training threshold was assessed by a ramp test where the exercise intensity on the bicycle ergometer was progressively increased by 25 watts in periods of 2 min at a constant pedaling frequency of 60 per min. Lactate level, pulse, blood pressure and wattage were recorded for each 2-min block. According to the results, maximal heart rate and wattage in aerobic threshold could be determined.

### Decompression chamber

The training for group III was conducted in a chamber with two connected generators (*Everest Summit Hypoxico Generator, Hypoxico Inc., New York, USA*), that filtered the room air and enriched the room air accordingly with nitrogen. O_2_ was reduced to the desired altitude. O_2_ concentration was 16–16.4% (equivalent to 2000 m above sea level) on the first three training days and 15–15.4% (equivalent to 2500 m above sea level) on consecutive training days. O_2_ and CO_2_ were monitored during the training. CO_2_ concentration was permanently below 1%. Each patient exercised 60 min per day under hypoxic conditions during the study period.

### Measurements

Fatigue was assessed at baseline, after 7 days and at the end of the study (14 days) using two questionnaires [Würzburger Erschöpfungsinventar bei Multipler Sklerose (WEIMuS) and the Modified Fatigue Impact Scale (MFIS)]. The WEIMuS is a validated self-assessment tool for quantifying fatigue and consists of two sub-domains, cognitive and physical, with a total of 17 items rated from 0 to 4, resulting in a total score of 0–68; fatigue was when the total scale value was ≥ 32 points [5, 6]. The MFIS has been proposed by the Multiple Sclerosis Council for Clinical Practice Guidelines as quantifying tool for measuring fatigue. The MFIS consists of three sub-domains, physical, cognitive, and psychosocial function. There are a total of 21 items with a score from 0 to 4, and the total score ranges from 0 to 84, with fatigue being defined from a value of ≥ 38 points [4]

Self-reported depressive symptoms were quantified by the Allgemeine Depressionsskala (ADS) which is the German validated and adapted Center for Epidemiologic Studies—Depression Scale (CES-D) [22].

Spasticity was evaluated using the Multiple Sclerosis Spasticity Scale 88 (MSSS-88), a patient-reported tool consisting of 88 items and 8 sub-domains. Each item can be scored from 0 to 4 with a total score ranging from 88 to 352 points [9, 10].

Walking endurance was quantified by the Six-Minute Walk Test (6MinWT) at the beginning and at the end of the study, which quantified the distance a participant could walk in 6 min when walking as fast as possible.

### Statistical analysis

Statistical analysis was performed with IBM SPSS 20.0.0 (SPSS Inc, Chicago, IL, USA) and GraphPad Prism® v6.01. For statistical analysis, data were used from all patients who completed at least 60% of the treatment protocol and who took part in all three examinations (baseline, 7 days, 14 days). Unless otherwise noted, data are given as mean (M) ± standard deviation (SD). Normal distribution was checked with the Kolmogorov–Smirnov test. Normality was analyzed with a one-factorial ANOVA. For data that were not normally distributed, non-parametric tests were used, as the Friedman and Wilcoxon test to determine differences between two points in time and, the Kruskal–Wallis and Mann–Whitney *U* Tests to compare the groups at a given time point. An intention-to-treat analysis was performed for all statistically significant changes in the per-protocol analysis.

A *p* value ≤ 0.05 was considered statistically significant (*), whereas *p* ≤ 0.01 was considered highly significant (**).

## Results

Characteristics of the study cohort are given in Table 1. The mean age was 43.2 ± 7.8 years, more females than male were enrolled (29:10), and median EDSS was 4 (1–6.5). Thirty-two patients participated in at least 60% of the training program and were thus used for further analysis (Fig. 1). Three patients of the ergometer group and four patients of the hypoxia group terminated the study prematurely before completing 60% of the tasks. Termination was mainly due to leg pain (n = 4) and an unusually long recovery period after the training (*n* = 3). The patients who withdrew from the study continued to receive the individually tailored rehabilitation program and took part in all measurements. Overall, 32 patients were included in the final analysis: 11 in the control group, 8 in the ergometer group, and 13 in the hypoxia group (Fig. 1). Regarding baseline differences in the respective study groups (*n* = 32), the control group and the ergometer group differed significantly (*p* = 0.012) in the disease duration in favor of the ergometer group, but no other variable differed between groups.

**Table 1:** Demographic and clinical characteristics of the entire cohort

	Control (*n* = 11)		Ergometer (*n* = 11)		Hypoxia (*n* = 17)	
Age^a^	43, 9	(6, 1)	41,5	(11, 3)	43,9	(6, 2)
Females^b^	9	(81, 8)	7	(63, 6)	13	(76, 5)
Disease duration^a^	14	(7)	6	(5)	13	(9)
EDSS^c^	6	(4,5)	3,5	(2,5)	4	(5, 5)
Barthel-Index^c^	100	(40)	100	(25)	100	(20)
ADSL-score^c^	14	(39)	15	(38)	10	(36)
DMT (*n*)	5		6		7	

^a^Mean and standard deviation

^b^Number (percentage)

^c^Median and range.

*EDSS* Expanded Disability Status Scale, *ADSL* Allgemeine Depressionsskala (German validated and adapted Center for Epidemiologic Studies—Depression Scale), *DMT* disease-modifying therapy

### Fatigue

At baseline, 24 patients on the WEIMuS and 22 patients on the MFIS scored more than 32 and 38 points, respectively, corresponding to fatigue. There was no difference between WEIMuS and MFIS scores between the groups at baseline. In the whole group, fatigue improved significantly after 2 weeks as measured by the WEIMuS or MFI overall scale and the subscales as well compared to baseline (*p* < 0.001; *p* < 0.001).

**Fig. 2:**
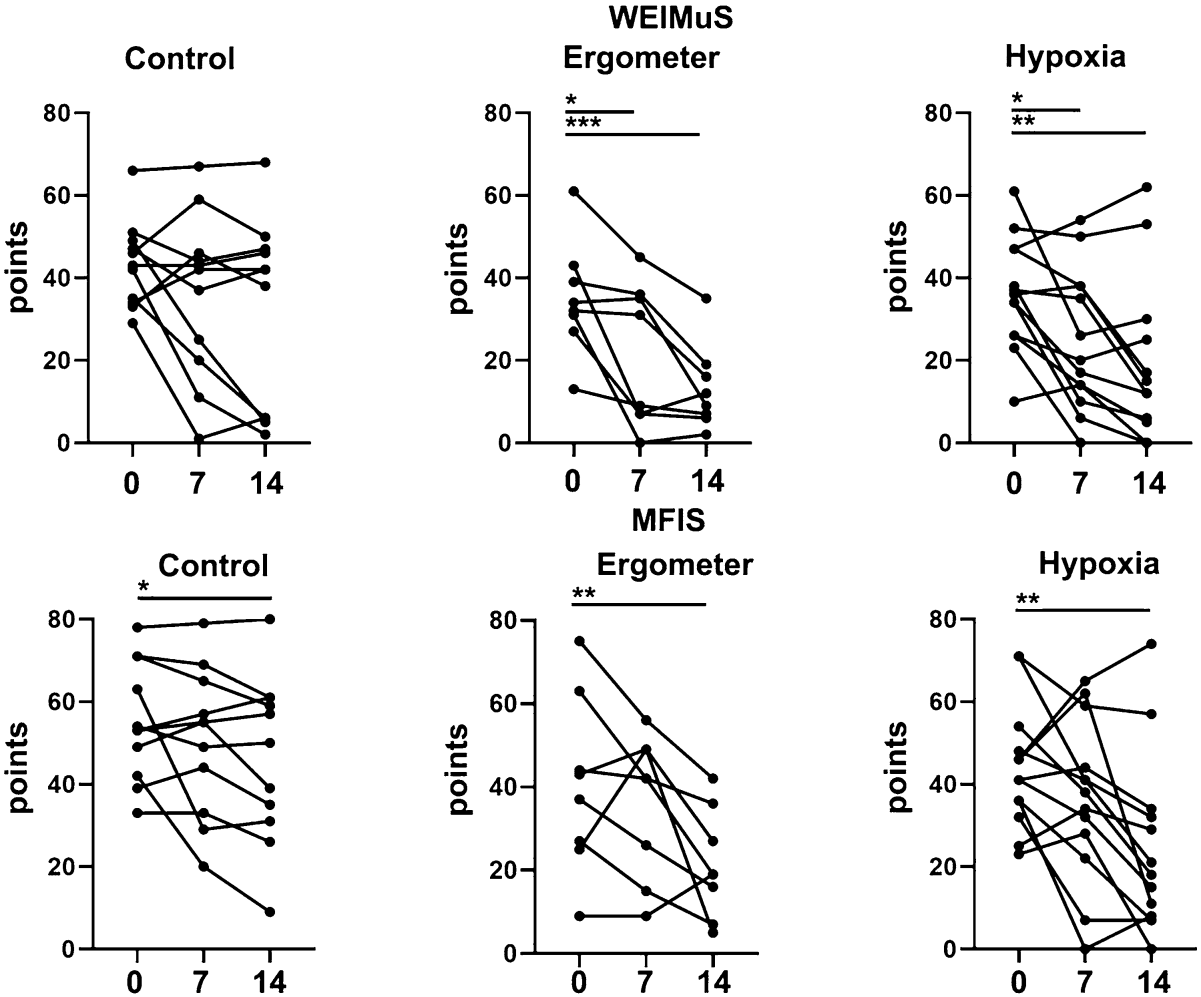
Comparison of fatigue scales

After 1 week of therapy, there was a significant improvement in the WEIMuS score in the ergometer (*p* = 0.03) and hypoxia groups (*p* = 0.01), but not in the control group (Fig. 2). After 2 weeks, all three groups showed significant improvements from baseline on the MFIS (control *p* = 0.049; ergometer *p* = 0.004; hypoxia *p* = 0.002), however, only the ergometer and hypoxia groups reached significantly fewer points on the WEIMuS scale (*p* < 0.001; *p* = 0.001; respectively).

Assessing differences between groups at day 14, the ergometer and hypoxia group showed significantly lower MFIS scores than the control group (*p* = 0.029; 0.027; respectively), while there was no difference in the WEIMuS score.

When performing the ITT analysis, all differences remained statistically significant except the differences between groups at day 14 which did not reach statistical significance.

### Mobility and spasticity

The overall group (*n* = 32) showed a significant improvement from baseline to day 14 (*p* < 0.001 in the 6MinWT and the MSSS-88, *p* = 0,002). At baseline, there was a significant difference in the 6MinWT between the control and the ergometer group (*p* = 0.008), otherwise, there was no difference between the groups.

**Fig. 3:**
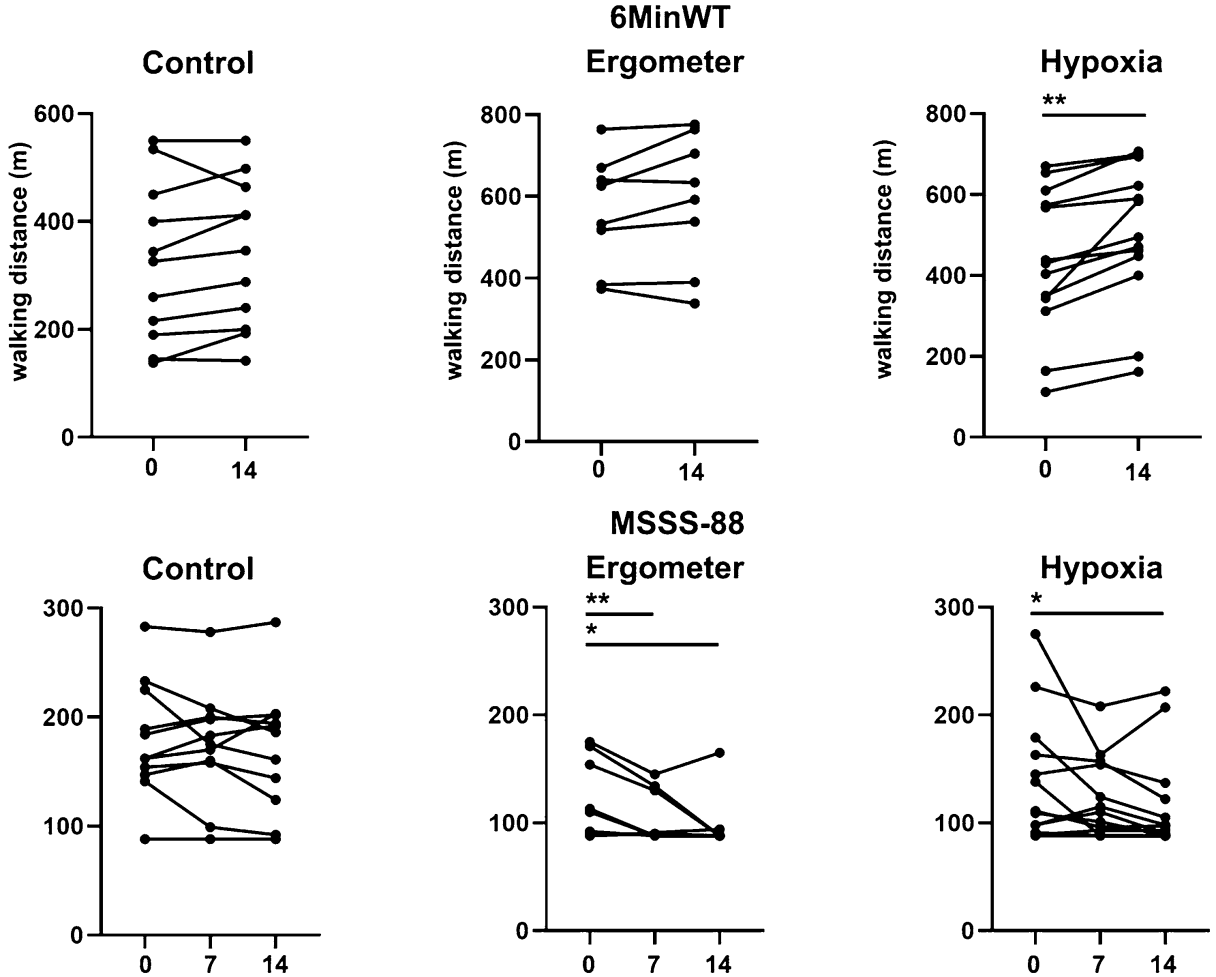
Comparison of mobility and spasticity

Patients in the hypoxia group improved significantly on the 6MinWT (*p* = 0.001) and reported lower impairment due to spasticity (*p* = 0.012) after 2 weeks compared to baseline (Fig. 3). In the ergometer group, significant improvement on the MSSS-88 scale was seen after 1 week (*p* = 0.009) and at the end of the study (*p* = 0.048). No significant improvement was seen in the control group.

Assessing differences between groups at day 14, the ergometer and hypoxia groups showed significant lower MSSS-88 scores compared to the control group (*p* = 0.007; 0.028; respectively) and the ergometer group achieved a significantly longer walking distance in the 6MinWT (*p* = 0.007) compared to the control group. When performing the ITT analysis, all described changes remained statistically significant.

## Discussion

In this single-blind prospective study, we provide evidence of a positive impact of endurance exercises in general on MS-related fatigue and spasticity; moreover, we show improved endurance capacity as measured by walking distance, when training was performed under hypoxic training conditions.

Reduced fatigue as measured by the MFIS was seen in all three groups after participating in the rehabilitation program and those receiving additional endurance training under different conditions. In line with previous studies, participation in rehabilitation alone positively affected the MFIS [11]. Likewise, this effect has been reflected in the WEIMuS scale, although significance was not reached in the control group, possibly due to sample size. Thus, our data support the positive effects of rehabilitation on fatigue with potentially additive effects associated with endurance training.

There is considerable evidence indicating the positive effects of endurance training on self-reported fatigue [8].We also found a significant improvement in fatigue already 1 week after training, and at the end of the study both endurance groups scored significantly lower on MFIS than the control group reflecting a lower degree of fatigue. However, we did not find an additional effect for training und normobaric hypoxic conditions on self-reported fatigue measurements compared to non-hypoxic conditions. In contrast, a previous study examining the effect of endurance training under hypoxic conditions did not report a beneficial impact on fatigue severity measured by the MFIS. These discrepancies are possibly explainable by the higher percentage of non-fatigued MS patients included in that study [13].

There is some evidence that physical activity programs improve spasticity in adult MS patients [1, 18]. We did find reduced self-reported impact of spasticity in both groups receiving additional endurance training, while there was no significant impact in the group which solely participated in the rehabilitation program. Further, both groups reported significantly less spasticity compared to the control group. Therefore, additionally, endurance training seems to positively impact self-reported spasticity although the underlying mechanism remains speculative. There was no additional benefit for spasticity observed in performing endurance training under hypoxic conditions.

In general, there is substantial evidence for exercise training on walking speed and endurance in MS patients [16]. Here, we used the 6-min walking test as a reliable measure of walking endurance which reflects reliable the functional exercise level for daily physical activities [2]. In contrast to previous studies, we only found a significant improvement in walking distance in patients performing endurance training under hypoxic conditions [12, 13, 23]. This may eventually be explained by the fact that our intervention only lasted 2 weeks, while most of the studies were designed for a considerably longer interval [13, 23]. Therefore, one could speculate that the hypoxic stimulus could have a positive effect on endurance performance.

The main strength of this study was the prospective study design, the three different patient subgroups, and the assessment of fatigue through various validated questionnaires. A major limitation of this study is the small sample size, the heterogeneous number of patients within the groups, and the limited intervention time, which may have constrained our conclusions and contributed to the exploratory nature of this study. In addition, we have no clinical or paraclinical follow-up to support the long-term benefits of this intervention.

Still, this study is one of the largest studies investigating the effects of normobaric hypoxic training in comparison to normoxic training and only rehabilitative activities in MS [13]. Although the study duration was only 14 days, this seems a feasible and reasonable duration to be implemented into a routine rehabilitation program of MS patients. Follow-up studies are needed to determine the long-term benefits of this intervention.

In conclusion, our findings demonstrate that endurance training may have an additional benefit to neurological rehabilitation programs in improving fatigue and the experience of spasticity. Furthermore, endurance training under hypoxic training conditions might positively impact walking endurance within a 2-week training intervention.
